# Virtual reality: A game-changing method for the language sciences

**DOI:** 10.3758/s13423-019-01571-3

**Published:** 2019-02-07

**Authors:** David Peeters

**Affiliations:** 1 Department of Communication and Cognition, Tilburg University, P.O. Box 90153, NL-5000 LE Tilburg, The Netherlands; 2 Max Planck Institute for Psycholinguistics, Nijmegen, The Netherlands; 3 Donders Institute for Brain, Cognition and Behaviour, Radboud University, Nijmegen, The Netherlands

**Keywords:** Virtual reality, Ecological validity, Psycholinguistics, Multimodal communication

## Abstract

This paper introduces virtual reality as an experimental method for the language sciences and provides a review of recent studies using the method to answer fundamental, psycholinguistic research questions. It is argued that virtual reality demonstrates that ecological validity and experimental control should not be conceived of as two extremes on a continuum, but rather as two orthogonal factors. Benefits of using virtual reality as an experimental method include that in a virtual environment, as in the real world, there is no artificial spatial divide between participant and stimulus. Moreover, virtual reality experiments do not necessarily have to include a repetitive trial structure or an unnatural experimental task. Virtual agents outperform experimental confederates in terms of the consistency and replicability of their behavior, allowing for reproducible science across participants and research labs. The main promise of virtual reality as a tool for the experimental language sciences, however, is that it shifts theoretical focus towards the interplay between different modalities (e.g., speech, gesture, eye gaze, facial expressions) in dynamic and communicative real-world environments, complementing studies that focus on one modality (e.g., speech) in isolation.

## Introduction

Natural languages are evolutionarily designed for face-to-face interaction (Levinson, 1983) and much of our everyday talk takes place in dynamic, communicative, audio-visual, 3D environments. Moreover, everyday communication is multimodal, in that we express our thoughts and intentions through multiple modalities such as speech, gestures, eye gaze, and facial expressions (Perniss, 2018). The experimental study of the cognitive and neural underpinnings of human linguistic and communicative capacities, however, often occurs in strictly controlled static, non-communicative lab settings in which unimodal stimuli are commonly presented to individual participants, out of context, via headphones or in 2D on a small computer monitor. The cognitive architecture supporting spoken language comprehension, for instance, is often studied by presenting individual participants with sequences of unrelated pre-recorded spoken words or sentences in the physical absence of both an actual speaker and a realistic, immediate, non-linguistic, and multimodal visual context. Not surprisingly, such "passive spectator science" (Hari, Henriksson, Malinen, & Parkkonen, 2015) has led to dominant neurocognitive theories of language comprehension that are highly language-centric and thereby do not do justice to the multimodal richness and dynamics of everyday communication (Knoeferle, 2015).

Undoubtedly, having strict experimental control has clear benefits, as it provides the researcher with the opportunity to make inferences about the role of a specific variable of interest in a particular process. For instance, when one presents a large group of participants with two sets of stimuli that are perfectly matched except for one variable (e.g., word valence) and finds a difference in response (e.g., as reflected by reaction times or brain activity) to the two sets, one can be reasonably sure that one’s variable of interest plays a role in the processing of one’s stimuli. A large discrepancy between the natural habitat of a phenomenon of interest and the experimental test setting, however, questions the ecological validity of obtained research findings and thereby the robustness and pertinence to everyday situations of subsequently generated theories (De Ruiter & Albert, 2017; Willems, 2015). The current paper introduces the unique potential for the language sciences of a relatively novel experimental method, *virtual reality*, which is argued to be capable of combining high ecological validity with high experimental control. Furthermore, this paper provides a review of recent experimental studies making use of virtual reality to study language processing in immersive three-dimensional settings.

## The interplay between ecological validity and experimental control

**Fig. 1:**
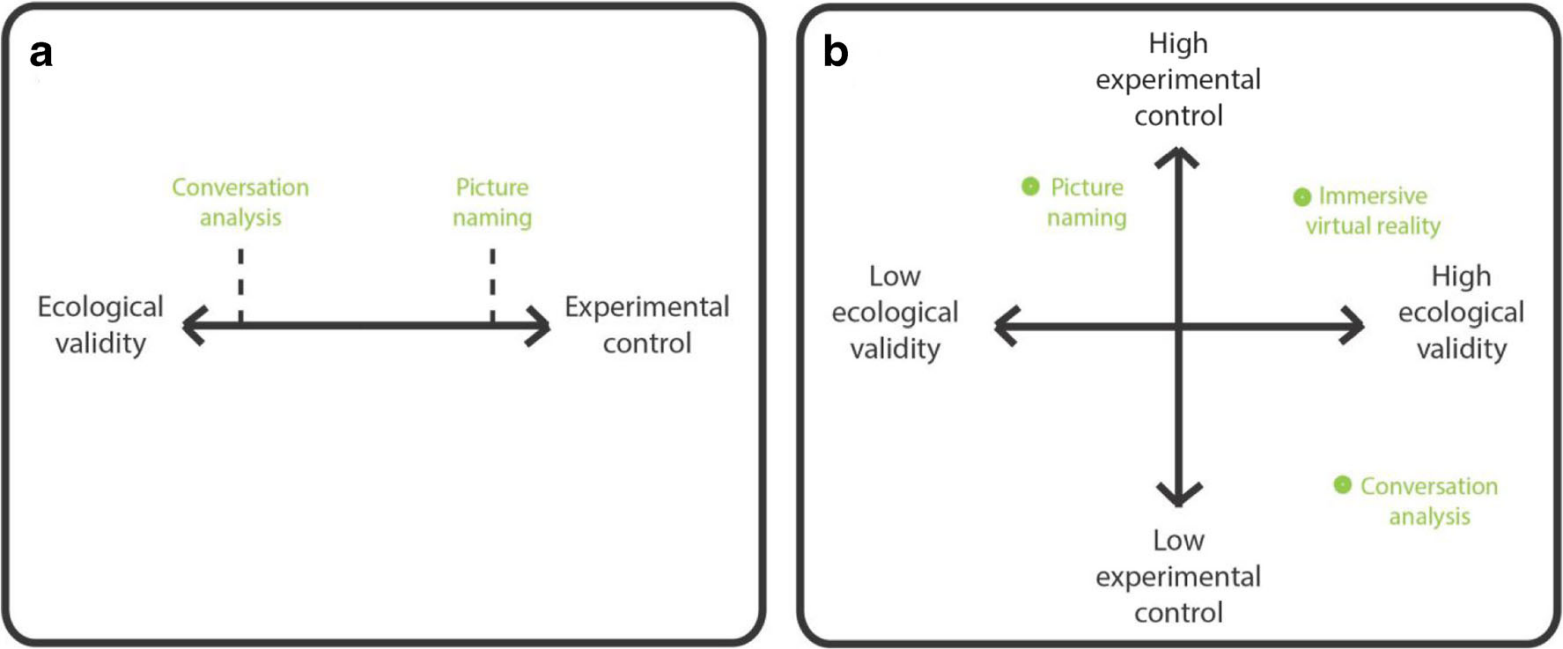
Ecological validity and experimental control in the language sciences perceived as two extremes on a continuum (**Panel A**) or as two orthogonal factors (**Panel B**)

Ideally, as a researcher experimentally investigating the psychology and/or neurobiology of language, one would want to combine solid experimental control with high ecological validity in one’s experimental study. At first sight, however, this seems impossible as those two constructs are commonly conceived of as two extremes on a single continuum (Fig. 1 panel A). It indeed appears straightforward to assign to the different methods and paradigms that we use in the language sciences a place somewhere on this continuum. Commonplace psycholinguistic experimental paradigms such as picture naming ignore much of the richness of everyday communication, but offer the researcher high levels of experimental control. A method like conversation analysis, on the other hand, respects the dynamics of everyday placed communication, but proffers the researcher much less control over the behavior of the observed participants. Other psycholinguistic paradigms, such as director-matcher tasks and certain variants of the visual world paradigm, might be placed somewhere in the middle. The existence of this one-dimensional continuum implicitly justifies the use of experimental methods that do not necessarily resemble everyday communication. Why do you have a psychology student tediously name 240 pictures presented one by one on a computer monitor in a soundproof booth in the absence of an addressee? Because language production in the “wild” is too noisy to provide you with reliable information about the potentially unique role in the speech production process of that one variable (e.g., word frequency) you are interested in.

Here it is argued that recent developments in virtual reality technology and their preliminary usage in the language sciences show that this continuum (Fig. 1 panel A) is misleading. Initial studies making use of virtual reality in the language sciences suggest that experimental control and ecological validity should be seen as two orthogonal factors instead (Fig. 1 panel B), as the method allows for experimental designs that combine the two constructs in an unprecedented way. By acknowledging this paradigm shift, significant progress in understanding the cognitive and neural basis of our human linguistic and communicative capacities *as active under everyday real-world circumstances* can now be made. By using and further developing available virtual reality methods it will be possible to strictly experimentally study brain and behavior in settings that nevertheless acknowledge the richness of everyday communication. Indeed, current-day virtual reality technology allows one to create three-dimensional virtual environments that mimic the complexity and dynamics of everyday situations while maintaining the experimental control necessary to collect reliable behavioral and neurophysiological data (Tromp, Peeters, Meyer, & Hagoort, 2018).

## Benefits of virtual reality for the language sciences

The conceptual difference between traditional experimental studies and virtual reality experiments is enormous. Instead of looking at stimuli one by one on a computer monitor, participants in virtual reality are immersed into a 3D environment. Rather than being passive observers of individual stimuli on a small computer screen, they enter the depicted scenes themselves. In other words, the artificial spatial divide between stimulus and participant disappears. As in the real world, participants are in the same space as the stimulus. In a sense, they are *in the stimulus*, as if they would jump into a picture or video presented on a computer screen in a traditional study and could interact with the content of that picture or video from within. Whereas computer monitors have intrinsic physical limitations in representing the dynamics, interactivity, and multimodal richness of everyday communication, the virtual realm allows for creating a world as dynamic, interactive, and rich (or even richer) as the real world. Furthermore, virtual reality experiments not necessarily contain the artificial trial structure that is present in typical psychological experiments. As in the real world, events of interest do not necessarily have to follow one another in a strictly timed and repetitive manner. Finally, in an average virtual reality experiment, unlike in traditional computer experiments, no additional artificial task (e.g., a response to “catch trials” or a metalinguistic judgment) is necessary to keep participants engaged and awake. We know that people’s metalinguistic intuitions often do not match their actual linguistic behavior (Clark & Bangerter, 2004).

**Fig. 2:**
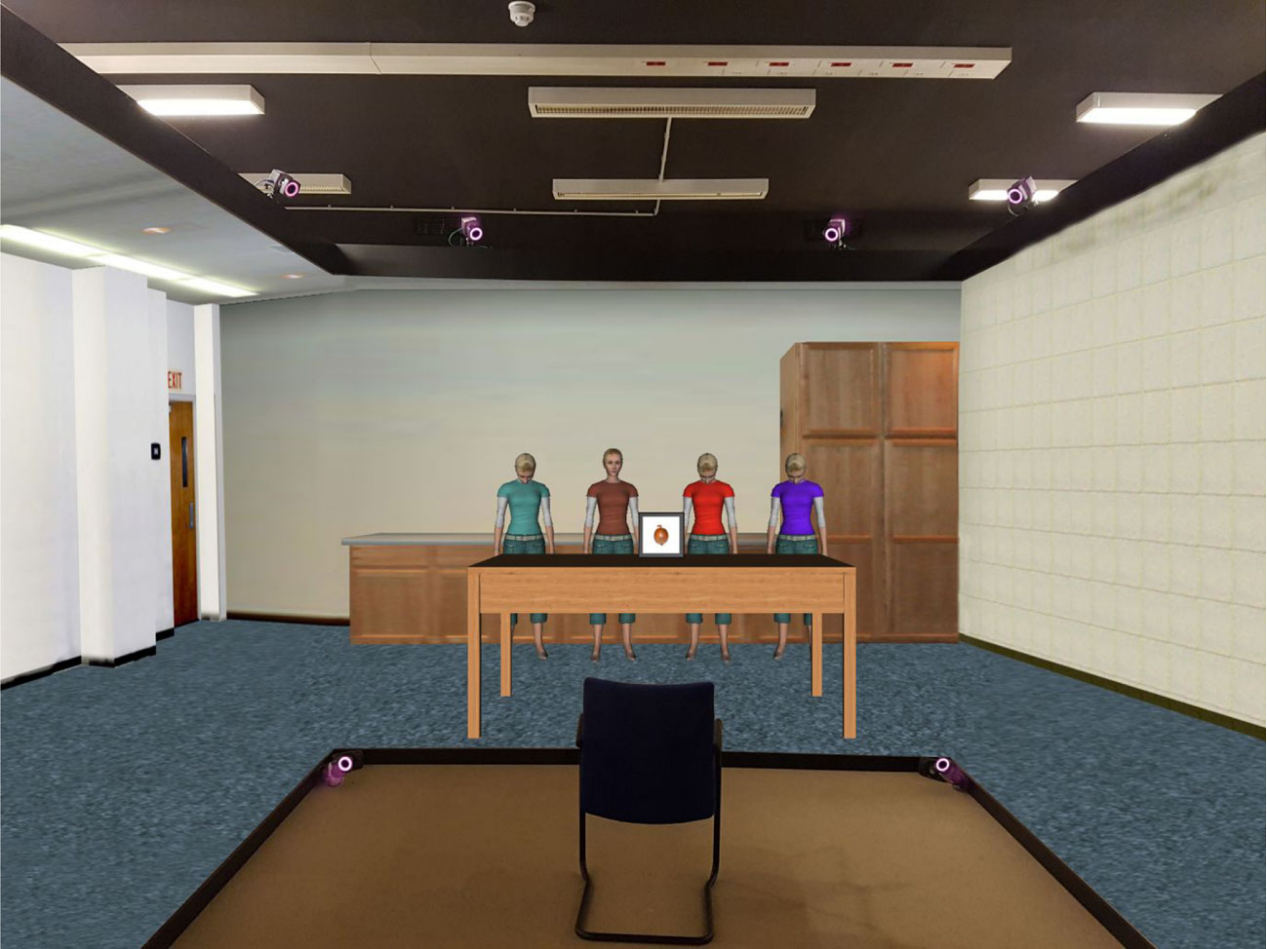
Example of a CAVE setting in which an experimental participant can be immersed into a virtual environment to interact with virtual objects and avatars. Participants wear shutter glasses that present the virtual world in 3D. Infrared cameras continuously track the position of the glasses to align the virtual environment with the gaze position of the participant

How is virtual reality different from traditional research methods in the language sciences at a practical level? In virtual environments, participants' eye, head, and body movements can be tracked and their digital surroundings rendered accordingly, typically via large projection screens or headsets in combination with a tracking system (see Fox, Arena, & Bailenson, 2009). This allows researchers to immerse participants in rich, customized settings that resemble real-world contexts while maintaining control over all the (visual, auditory, haptic, olfactory, and in principle also gustatory) sensory input the participant receives. In CAVE setups (Fig. 2), participants are surrounded by large projection screens or walls on which dynamic virtual content is presented and adapted on-line to the viewpoint of the user (Cruz-Neira, Sandin, & DeFanti, 1993). By wearing shutter glasses, they become immersed in the virtual environment while still being able to see their own body. In contrast, virtual reality headsets (or: head-mounted displays) such as Oculus Rift or HTC Vive almost completely replace real-world input by a virtual alternative. CAVE setups may be preferred when the researcher is interested in recording brain activity through near-infrared spectroscopy (NIRS) or electroencephalography (EEG, but see Peeters & Dijkstra, 2018; Tromp et al., 2018) and when wishing to objectively analyze participants’ hand gestures or facial expressions. The use of headsets is less expensive and will typically lead to a higher degree of experienced presence in the virtual environment. Both CAVE setups and headsets can be combined with eye tracking.

Of course, in the past other attempts have been made to improve ecological validity in experimental lab settings, and not all lab experiments in the language sciences are restricted to a participant performing a repetitive, individual task on a computer. Paradigms have been developed, for instance, in which a participant interacts with a confederate who is trained to behave in a consistent and relatively naturalistic way while still allowing for an experimental test of a theoretically motivated variable of interest. Virtual reality offers an alternative to such approaches that bypasses many of the problems that arise when one uses confederates in experimental research (see Kuhlen & Brennan, 2013). Whereas confederates simply cannot behave in the exact same way with every experimental participant, virtual agents can be programmed to do so. By using the same virtual reality scenario with the same consistently-behaving virtual agent between participants, within and across different experimental labs, the direct reproducibility of an experimental finding can now be assessed more reliably than before (Pan & Hamilton, 2018).

Whereas an experiment’s degree of experimental control and a finding’s reproducibility can be determined in a relatively straightforward manner, its ecological validity is more difficult to quantify. So what is the evidence that virtual reality offers experimental studies a higher degree of ecological validity than traditional studies? In principle, there will rarely be direct evidence for this claim, as the degree of ecological validity of a specific study often remains an assumption. If it were possible to directly test an experiment’s ecological validity in the real world, it would not be necessary to conduct that experiment in the lab. However, a reasonable approach in this matter seems to be to classify the attributes most central to the real-world phenomenon one is interested in and verify whether these are present in one’s experiment (e.g., Schmuckler, 2001). Critical aspects of typical everyday communication, such as that it usually involves an actual speaker (or signer) and at least one addressee, that it is an intrinsically multimodal activity (comprising information transmitted via speech, gesture, facial expressions, etc.) and takes place in dynamic, 3D environments, are more easily represented in virtual reality settings than in typical traditional experiments. In cases where an experiment can be carried out similarly in the real world and in a virtual environment, sometimes the virtual alternative might even be the preferred option. For instance, if one is interested in experimentally studying public speaking, using an audience of virtual agents might be more feasible than finding a large group of human volunteers (Slater, Pertaub, Barker, & Clark, 2006).

Finally, researchers interested in the influence of individual differences on language processing might also benefit from the availability of virtual reality. It has been argued that standard neuropsychological tests of individual traits (e.g., working memory capacity, attention span) using paper-and-pencil assessment and/or static stimuli may have limited ecological validity in that their results may not be representative of individuals’ real-world functioning (Chaytor, Schmitter-Edgecombe, & Burr, 2006; Parsons, 2015). As an alternative, tests of individual differences may be carried out in virtual environments resembling the real world (e.g., Renison, Ponsford, Testa, Richardson, & Brownfield, 2012) before being correlated with measures of language processing and behavior.

## Virtual reality in psycholinguistics: A review of recent studies

Initial studies that have used virtual reality as a method in psycholinguistics over the last decade can be divided into two categories. A first line of studies have tested whether well established findings from traditional studies replicate in rich virtual environments. It has been found, for instance, that language-driven anticipatory eye movements to objects are observed in a virtual setting similar to traditional psycholinguistic eye-tracking paradigms (Eichert, Peeters, & Hagoort, 2018). The proportion of passive constructions that people use increases as much when they are primed in a dialogue by a 3D virtual human-like partner compared to when they are primed by a human partner (Heyselaar, Hagoort, & Segaert, 2017a). When bilinguals switch languages between virtual interlocutors with different language backgrounds, similar behavioral and neural switch costs are observed compared to traditional cued-switching paradigms (Peeters & Dijkstra, 2018). Finally, when virtual agents in a rich visual environment refer to an object using an incorrect label in speech, a robust and widespread N400 effect is found compared to when they correctly refer to the object – an effect very similar to the N400 effect induced by such mismatches in traditional non-virtual, 2D approaches using speech and static pictures of a human agent referring to an object (Tromp et al., 2018). Hence, when using similar manipulations, virtual reality paradigms yield similar results compared to well established traditional paradigms. These initial results thereby validate virtual reality as a reliable experimental method and confirm the feasibility of using behavioral measures, eye tracking, and EEG in virtual environments. Although they may indicate whether traditional findings have any real-world value, these findings do not necessarily show the added value of virtual reality compared to other experimental methods at a theoretical level.

A second type of psycholinguistic studies have made use of virtual reality as a method to carry out experiments that are hard or even impossible to do in the real world. It has been observed, for example, that people accommodate their pitch and their speech rate to the pitch level and the speech rate of their virtual interlocutor (Gijssels, Casasanto, Jasmin, Hagoort, & Casasanto, 2016; Staum Casasanto, Jasmin, & Casasanto, 2010). Having a human interlocutor across different conditions keeping all aspects of their behavior constant except for their pitch or speech rate is practically impossible, but virtual agents can be programmed to do so. The same holds when one is interested in testing the effect of specific non-verbal habits such as smiling and eye-blink rate and their social consequences for language processing (Heyselaar et al., 2017a; Heyselaar, Hagoort, & Segaert, 2017b; Hömke, Holler, & Levinson, 2018). These studies illustrate that virtual reality allows for a test of the unique and potentially causal contribution of a single variable of interest (e.g., speech rate, pitch, smiling, blink rate) on aspects of language production and perception. Moreover, it does so in naturalistic environments that resemble everyday communicative situations, such as when talking to someone in a virtual supermarket (Gijssels et al., 2016) or when playing a (virtual) card game with somebody (Heyselaar et al., 2017a).

Furthermore, in the study of the synchronization between different communicative modalities, such as concurrent speech and gesture, virtual reality has proven a valuable method. Specifically, it has been found that virtually disrupting the visual feedback of participants’ pointing gesture trajectory affected concurrent speech production, which shows that gesture and speech production mechanisms continuously interact during the production of multimodal messages (Chu & Hagoort, 2014). In this specific domain, virtual reality is undoubtedly a methodological step forward compared to earlier experiments in which participants’ pointing gestures were disrupted during their execution by a mass applied to the participant’s wrist via a cord attached to it (Levelt, Richardson, & La Heij, 1985).

Another illustrative example of how virtual reality goes beyond traditional experimental methods comes from the field of indirect speech processing (Tromp, 2018). Earlier experimental studies in this domain commonly presented participants with short written scenarios on a computer screen, removing the speech acts from their typical, everyday contexts. By building a virtual restaurant in which participants were waiters and encountered customers that indirectly complained about their food, it was proven possible to take the controlled, experimental study of indirect speech back into a virtual equivalent of its complex, natural habitat (Tromp, 2018). A traditional experimental study in this domain might have asked participants to *imagine* being a waiter in a restaurant before listening to an indirect speech act like “my soup is cold” via headphones. In a virtual restaurant, participants can see themselves reflected as a waiter in a virtual mirror before encountering a virtual customer that directly addresses them by looking them in the eyes and indirectly complaining about the food. Preliminary findings suggest that the additional processing cost for indirect speech acts (“my soup is cold”) compared to direct statements (“my soup is nice”), typically observed in traditional studies in this domain, disappears when the participant is immersed in a rich, everyday context (Tromp, 2018). As such, traditional experimental studies in the language sciences may have shown us what the brain *can* do, not what it *will* do, in everyday communication.

Together, these initial studies using virtual reality as a method to answer psycholinguistic questions allow for some first conclusions on how to best use the method. An approach that has not necessarily led to ground-breaking theoretical advances is one in which one starts from an existing experimental paradigm and aims to develop a virtual equivalent of it. Typically, in such cases, the traditional and the virtual paradigm will lead to the same outcome. Picture naming in virtual reality does not add much compared to picture naming on a computer screen. Rather, one should start from the research question. If one’s topic of scientific interest is intrinsically multimodal and rich in nature, one wishes to study it experimentally, and considers it important to generalize one’s results to everyday situations, setting up a virtual reality experiment is the way to go.

Current limitations of virtual reality as a method for the language sciences mainly relate to the (lack of) spontaneity in bi-directional human-agent interactions. It is possible for a human participant to have a relatively naturalistic conversation with a virtual agent by using a *Wizard-of-Oz* procedure in which the experimenter (the “human-in-the-loop”) selects the agent’s contextually appropriate response from a selection of pre-defined (multimodal) response options (e.g., Pan et al., 2016). However, such conversations often follow a narrow script and the number of potential responses that a virtual agent can provide is still limited in the light of the human capacity to combine a finite number of linguistic building blocks in a virtually infinite way. Another challenge lies in extending the technological possibilities to track and render on-line the fine details of a person’s non-verbal behavior (e.g., the subtle details in a person’s hand gestures or facial expressions) onto that person’s avatar in a virtual environment when he or she is wearing a head-mounted display. Finally, care should be taken when using virtual reality as a research method to study language processing in children, as they may develop false memories and may have difficulties cognitively dissociating the virtual realm from the real world (Segovia & Bailenson, 2009).

## Conclusions and outlook

This paper argued that virtual reality provides a unique combination of experimental control, ecological validity, and reproducibility (cf. Blascovich et al., 2002; Casasanto & Jasmin, 2018; Pan & Hamilton, 2018), rendering it a potentially game-changing method for the language sciences. At the conceptual level, virtual reality will lead to a paradigm shift in that it overcomes the artificial gap between participant and stimulus and removes the need for a repetitive trial structure from experimental studies. Initial studies using virtual reality in the language sciences have shown that one can collect data as reliably in virtual reality as when using traditional methods. Moreover, the method has been applied successfully in subfields of the language sciences as diverse as indirect speech processing, syntactic priming, predictive language processing, multilingualism, and gesture studies. Perhaps the main promise of virtual reality as a research tool for the experimental language sciences is that it will shift theoretical focus towards the interplay between different modalities (e.g., speech, gesture, eye gaze, facial expressions) in dynamic and communicative real-world environments, moving beyond and complementing studies that focus on one modality in isolation.

This paper has focused on the use of virtual reality to answer *fundamental* research questions related to language. However, when it comes to combining fundamental with applied scientific interests, recent studies also show the potential of using the method. In the domain of foreign language acquisition, for instance, virtual reality has been successfully used to immerse learners of a second language into a congruent foreign setting in which they can socially interact with native speakers of the language they intend to acquire (see Lin & Lan, 2015, for overview). The full-time availability and portability of virtual interlocutors, and the fact that they can be tailored to the individual needs of the learner, make them a valuable new learning tool (cf. Macedonia, Groher, & Roithmayr, 2014). In the treatment of language-related disorders and phobia, virtual reality also offers novel possibilities. As an example of exposure therapy, virtual reality has, for instance, been applied with promising results to reduce public speaking anxiety by having participants practice their speeches in front of distracting virtual audiences (Slater, Pertaub, & Steed, 1999).

Finally, at a technical and practical level, there is no reason not to start making (more) use of virtual reality as a research method. Setting up a functioning virtual reality lab that uses a headset such as an Oculus Rift or an HTC Vive is no longer significantly more expensive than setting up an experimental lab that uses a computer monitor as its main medium of display. Designing three-dimensional virtual objects and environments requires graphic design and programming skills, but students in Data Science, New Media Design, and Artificial Intelligence have these skills. Alternatively, three-dimensional objects can easily be retrieved from online, standardized databases (Peeters, 2018). With these practical issues out of the way, the availability of virtual reality as a research method now offers the potential to do justice to the multimodal richness and dynamics of everyday communication in our cognitive theories of language.
